# Interruption of mitochondrial symbiosis is associated with the development of osteoporosis

**DOI:** 10.3389/fendo.2025.1488489

**Published:** 2025-02-03

**Authors:** Haoling Zhang, Rui Zhao, Xuemei Wang, Yaqian Qi, Doblin Sandai, Wei Wang, Zhijing Song, Qiudong Liang

**Affiliations:** ^1^ Department of Spinal and Trauma Surgery, The First Affiliated Hospital of Xinxiang Medical College, Xinxiang, Henan, China; ^2^ Department of Biomedical Sciences, Advanced Medical and Dental Institute, Universiti Sains Malaysia, Penang, Malaysia; ^3^ School of Public Health, Gansu University of Traditional Chinese Medicine, Lanzhou, China; ^4^ Clinical College of Chinese Medicine, Gansu University of Chinese Medicine, Lanzhou, China; ^5^ College of Pharmacy, Gansu University of Traditional Chinese Medicine, Lanzhou, China; ^6^ College of Acupuncture and Massage, Gansu University of Traditional Chinese Medicine, Lanzhou, China; ^7^ Key Laboratory of Dunhuang Medicine and Transformation, Ministry of Education, Lanzhou, China

**Keywords:** interruption of mitochondrial endosymbiosis, osteoporosis, cytoplasmic signal, bone tissue cells, heredity, ADAPT, targeted therapy

## Abstract

Mitochondria maintain bacterial traits because of their endosymbiotic origins, yet the host cell recognizes them as non-threatening since the organelles are compartmentalized. Nevertheless, the controlled release of mitochondrial components into the cytoplasm can initiate cell death, activate innate immunity, and provoke inflammation. This selective interruption of endosymbiosis as early as 2 billion years ago allowed mitochondria to become intracellular signaling hubs. Recent studies have found that the interruption of mitochondrial symbiosis may be closely related to the occurrence of various diseases, especially osteoporosis (OP). OP is a systemic bone disease characterized by reduced bone mass, impaired bone microstructure, elevated bone fragility, and susceptibility to fracture. The interruption of intra-mitochondrial symbiosis affects the energy metabolism of bone cells, leads to the imbalance of bone formation and bone absorption, and promotes the occurrence of osteoporosis. In this paper, we reviewed the mechanism of mitochondrial intersymbiosis interruption in OP, discussed the relationship between mitochondrial intersymbiosis interruption and bone marrow mesenchymal stem cells, osteoblasts and osteoclasts, as well as the inheritance and adaptation in the evolutionary process, and prospected the future research direction to provide new ideas for clinical treatment.

## 1 Introduction

Mitochondria serve a crucial function as the cellular powerhouses, with their optimal performance being indispensable for sustaining cellular health and ensuring the overall homeostasis of organisms. In recent years, research on mitochondria has progressively broadened to encompass their symbiotic interactions with other cellular entities, particularly their relationships with host cells (1). For example, Nitric oxide interrupts mitochondrial apoptosis signaling through S-nitrosation of caspase-8, thereby preventing tumor necrosis factor-α-induced apoptosis of rat hepatocytes (2). Bay 11-7082 blocks the NF-κB pathway and promotes UCN-01-mediated mitochondrial interruption and apoptosis in human multiple myeloma cells (3). Mitochondrial symbiosis refers to the interaction between mitochondria within the cell or between mitochondria and other organelles, and can describe cooperative exchange such as mitochondrial fusion, fission, or the exchange of metabolites between mitochondria and other organelles such as the endoplasmic reticulum (4, 5). Mitochondrial symbiosis will be limited to intracellular processes and interactions within the cells of individual organisms, rather than interactions between different organisms. Endosymbiosis involves one organism living inside the cells of another, such as how mitochondria are thought to have evolved. Mitochondrial symbiosis can describe the interactions between mitochondria or their relationships with other organelles within the cell. Unlike symbiosis, which involves two different organisms, or endosymbiosis, in which one organism lives in another, mitochondrial symbiosis would be limited to intracellular processes and interactions within the cells of a single organism, rather than interactions between different organisms (6, 7).

The disruption of mitochondrial endosymbiosis refers to the breakdown of the evolutionary mutual dependence between mitochondria and the host cell, which originally allowed for the integration of free-living bacteria into a symbiotic organelle. This disruption can lead to the loss of key mitochondrial functions or extensive degradation of the mitochondrial genome, ultimately causing a significant decline in mitochondrial efficiency within the host cell (8). Mitochondria, which evolved from primitive bacteria via endosymbiosis, have transferred many of their genetic functions to the host cell’s nucleus over time. However, when this process is interrupted—whether by environmental stressors, failures in gene transfer, or reduced cellular reliance on mitochondria—it results in severe impairment of mitochondrial activity.

The interruption of mitochondrial symbiosis, particularly in chronic pathological conditions, underscores its profound effect on cellular survival and overall physiological homeostasis. Such interruption leads to a substantial reduction in intracellular energy availability, directly weakening the cell’s capacity for proliferation and differentiation, especially in energy-demanding cell types like osteoblasts and myocytes (9). Furthermore, the interruption of mitochondrial symbiosis signifies an adaptive response to fluctuations in both internal and external environments affecting biological energy metabolism. In the face of environmental stressors, cells frequently depend on the adaptive modulation of mitochondria to preserve internal stability. However, when this adaptive mechanism degradation, cells may enter a state of “energy deficiency,” further exacerbating pathological damage (10).

The interruption of mitochondrial symbiosis primarily arises from a multitude of mechanisms, including oxidative stress, mutations in mitochondrial DNA, and the depletion of mitochondrial membrane potential (11–13). The heightened production of ROS (reactive oxygen species) induced by oxidative stress can cause damage on the mitochondrial membrane, impairing its functionality and consequently diminishing ATP synthesis (14). Concurrently, mitochondrial mutations may result in the dysfunction of the electron transport chain, further exacerbating metabolic disorders within cells (15). Additionally, a reduced mitochondrial membrane potential disrupts the internal storage and release of calcium, thereby impairing the normal operation of cellular signaling pathways (16). Collectively, these factors lead to in insufficient cellular energy, heightened apoptotic signaling, and ultimately, tissue damage.

Oxidative stress and inflammation caused by aging, combined with cellular damage caused by mitochondrial dysfunction, create a vicious cycle that severely impairs bone health. As the global population ages, the prevention and management of OP (osteoporosis) have become increasingly crucial. Osteoporosis is a metabolic bone disorder characterized by a reduction in bone mass and the deterioration of the microarchitecture of bone tissue (17). Its pathogenesis is intricately linked to mitochondrial dysfunction (18). Research indicates that the decline in mitochondrial efficiency directly inhibits the activity of both osteoblasts and osteoclasts. The energy deficiency experienced by osteoblasts is closely associated with dysfunctional mitochondrial function, leading to diminished proliferation, differentiation, and a reduce in bone matrix synthesis. Moreover, ROS generated by mitochondria play a dual role in bone metabolism; while moderate levels of ROS can promote osteoblast differentiation, excessive ROS production initiates osteoclasts, thereby increasing bone resorption (19). Recent studies have provided compelling evidence that the interruption of mitochondrial symbiosis is directly connected with the onset of osteoporosis. For instance, mitochondrial function is typically impaired in individuals with osteoporosis, and mitochondrial DNA content shows a significant positive correlation with bone density (20, 21). In animal models, enhancing mitochondrial function or inhibiting ROS production has been shown to effectively improve bone mineral density, suggesting that restoring mitochondrial function may represent a viable strategy for the prevention and treatment of osteoporosis (22).

In recent years, mounting evidence has indicated that the interruption of mitochondrial symbiosis and its interactions with host cells may significantly influence the regulatory mechanisms governing bone metabolism. Particularly during the aging process, the decline in mitochondrial function is closely linked to the pathogenesis of osteoporosis. This insight underscores the importance of conducting an in-depth examination of how mitochondrial symbiosis interruption inhibits bone metabolism, not only to elucidate the pathophysiological mechanisms underlying osteoporosis but also to generate innovative strategies for its prevention and treatment. In summary, this study aims to meticulously elucidate the association between the interruption of mitochondrial symbiosis and the progression of osteoporosis, with the intent of providing new perspectives on the mechanisms of related diseases and clinical interventions. By uncovering the intricate relationship between mitochondrial function and bone metabolism, we aspire to lay the groundwork for the development of future therapeutic strategies for osteoporosis.

## 2 The disrupted mitochondrial symbiosis conveys signaling mechanisms to the cytoplasm

**Figure 1:**
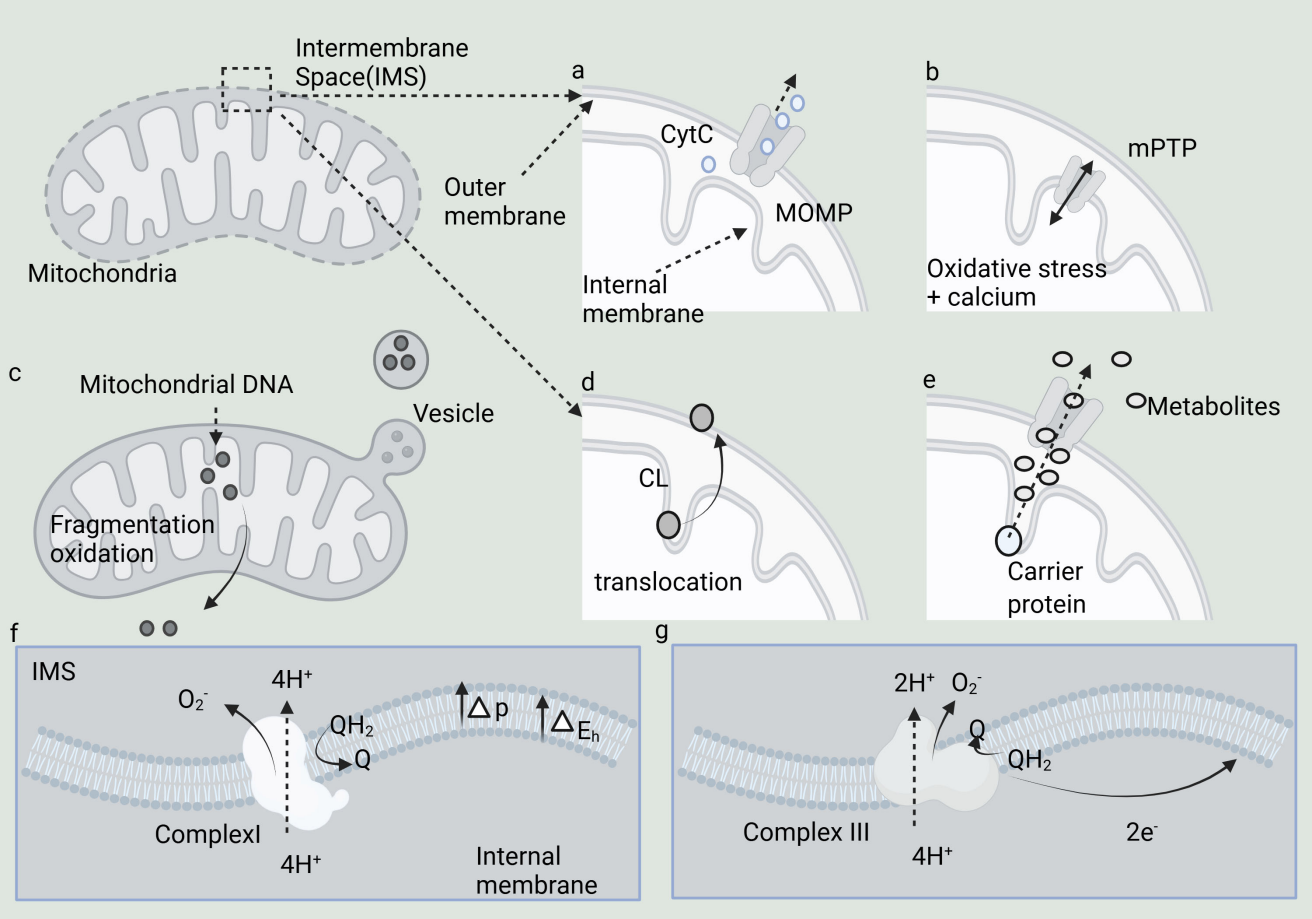
Mechanisms underlying the evolution of disrupted mitochondrial symbiosis and its signaling to the cytoplasm. **(A–G)** Seven key mechanisms.

This paragraph primarily explores the mechanisms of intracellular signal transduction resulting from the interruption of mitochondrial symbiosis, which encompasses seven key mechanisms (**Figure 1**). These mechanisms include: (a) MOMP (Mitochondrial outer membrane permeability), which enhances the permeability of the mitochondrial outer membrane, thereby facilitating the release of various essential proteins, such as Cytc (cytochrome c); (b) The opening of the MPTP (mitochondrial permeability transition pore), which promotes the efflux of Ca^2+^ ions and activates cell signaling pathways; (c) The release of mtDNA (mitochondrial DNA) into the cytoplasm, which triggers the expression of numerous pro-inflammatory genes; (d) CL (Cardiolipin), which plays a crucial role in regulating the curvature of the mitochondrial membrane; (e) Mitochondrial metabolites, including citric acid and succinic acid, that modulate the activity of specific protein targets; (f, g) Complexes I and III, which facilitate the directed transfer of protons into the mitochondrial intermembrane space.

The interruption of mitochondrial symbiosis implies that the delicate relationship between mitochondria and host cells is disrupted, triggering the activation of multiple cellular signaling mechanisms. Mitochondria are pivotal in immune signaling and programmed cell death, with their functional state directly influencing the intensity of immune responses and determining cell survival outcomes. Through various mechanisms, mitochondria can release molecules into the cytoplasm, thereby activating numerous immune signaling pathways or programmed cell death processes, highlighting the profound impact of mitochondrial “heterogeneity” on the rest of the cell (23, 24). MOMP is a critical event in apoptosis and programmed cell death (25, 26). MOMP leads to enhanced permeability of the mitochondrial outer membrane, allowing the release of various crucial proteins such as Cyt c, AIF, Smac/DIABLO, cytochrome b5, and mtDNA. These proteins play crucial roles in cell survival, apoptosis, and stress responses (27–29). The process of MOMP is controlled by the Bcl-2 protein family, which includes both pro-apoptotic proteins (like Bax and Bak) and anti-apoptotic proteins (such as Bcl-2 and Bcl-xL) (30). MOMP can be induced by both endogenous stimuli (such as DNA damage, oxidative stress) and exogenous signals (such as cytokines, drugs) (31).

The MPTP is a calcium-dependent, non-selective ion channel in the membrane that serves various functions. Despite over 50 years of research, its molecular structure remains elusive. MPTP spans both the inner and outer mitochondrial membranes, functioning as a non-specific channel for signal transduction and material transfer between the mitochondrial matrix and cytoplasm. It plays a key role in maintaining Ca^2+^ homeostasis, regulating oxidative stress signals, and mediating certain stimulation-induced protein translocations (32). The short-term (reversible) opening of MPTP protects cells from oxidative damage by promoting Ca^2+^ ion efflux from the mitochondrial matrix, facilitating cell signaling. mtDNA, which lacks CpG methylation, closely resembles bacterial DNA. When mtDNA is released into the cytoplasm, it is recognized by bacterial DNA sensors such as TLR9 and cGAS, triggering the expression of multiple pro-inflammatory genes (33–35).

The release of mtRNA (mitochondrial RNA) triggers viral sensing pathways via RIG-I-like receptors, which interact with MAVS (Mitochondrial Antiviral Signaling Protein) on the surface of the outer mitochondrial membrane. The N-formylation of mitochondrial translation polypeptides results in N-terminal N-formylated methionine residues, a hallmark of bacterial proteins that underscores the connection between mitochondria and their bacterial origin (8, 36, 37). CL plays a crucial role in regulating membrane curvature in both bacterial and mitochondrial membranes, and is vital for the function of various membrane proteins, which explains why mitochondria have retained CL. CL is found in the mitochondria of all eukaryotes and possesses a unique dimer structure, comprising two phosphatidic acid residues linked by glycerol bridges, which imparts distinct physicochemical properties (38). In the mitochondrial respiratory chain, complexes I and III are key sources of superoxide production and may play a role in mitochondrial redox signaling. Complex I (nicotinamide adenine dinucleotide, NADH) and Complex III (Ubiquinol: cytochrome c oxidoreductase; Cytochrome BC-1 complex) are thought to generate superoxide anions, which are released into the mitochondrial matrix and intermembrane space, respectively. The main role of these two complexes is to capture energy from redox reactions to facilitate the directional transfer of protons into the mitochondrial intermembrane space (39).

Mitochondrial metabolites, such as citric acid and succinic acid, function as signals by modulating the activity of protein targets or influencing DNA and histone methylation, thereby serving as potential epigenetic signals. Mitochondria orchestrate various metabolic pathways to generate metabolites essential for cell survival and proliferation. As a distinct metabolic hub, the TCA (tricarboxylic acid) cycle not only supports energy metabolism and the production of NADH to facilitate the respiratory chain but also provides metabolites with specialized signal transduction roles, such as acetyl-CoA, citrate, cis-aconitate, isocitrate, 2-oxoglutarate, succinate, and fumarate (40). The Warburg effect signifies the metabolic shift of cells from oxidative phosphorylation to glycolysis, without indicating a complete loss of mitochondrial function, as active mitochondria are still necessary in most tumor and inflammatory states.In this metabolic profile, referred to as the Warburg effect, most of the pyruvate generated from glycolysis is redirected toward lactic fermentation, circumventing mitochondrial oxidative metabolism (41).This section explores the complexities of intracellular signal transduction mechanisms precipitated by the interruption of mitochondrial symbiosis, encompassing seven pivotal processes. Each of these processes plays a crucial role in cellular responses, ranging from apoptosis to immune signaling and metabolic regulation.

## 3 Mechanisms of mitochondrial symbiosis destruction and osteoporosis

In this section, we will investigate the mechanisms behind the breakdown of mitochondrial symbiosis and its connection to osteoporosis. Specifically, we will examine how the interruption of mitochondrial symbiosis affects osteoblasts, osteoclasts, and bone marrow mesenchymal stem cells, leading to a decline in bone mineral density, increased bone resorption, and slowed regeneration of bone marrow mesenchymal stem cells. These factors collectively contribute to the worsening of bone loss. This exploration underscores the significant role of mitochondrial dysfunction in influencing bone health and advancing the progression of osteoporosis.

### 3.1 Interruption of mitochondrial endosymbiosis with osteoblasts

In recent years, the focus on mitochondrial endosymbiosis has expanded beyond its traditional role in bioenergetics, revealing its profound implications in various physiological contexts, particularly in bone biology. Osteoblasts, the bone-forming cells, rely heavily on mitochondrial function for their differentiation and activity. Interruption of mitochondrial endosymbiosis can lead to significant alterations in osteoblast function, potentially contributing to pathological conditions such as osteoporosis.

AOPPs (Advanced oxidized protein products) promote the generation of reactive oxygen ROS from NOX (nicotinamide adenine dinucleotide phosphate oxidase), resulting in the depolarization of the mitochondrial membrane potential (ΔΨm). This activates the mitochondria-dependent intrinsic apoptotic pathway, leading to osteoblast apoptosis, which contributes to osteopenia and deterioration of bone microstructure. PINK1/Parkin-mediated mitophagy reduces plasma AOPP levels, inhibits AOPP-induced osteoblast apoptosis, and mitigates bone loss, bone microstructure degradation, and the decrease in BMD (bone mineral density) associated with AOPP accumulation (42). Nitric oxide, released by sodium nitroprusside, induces osteoblast apoptosis through a mitochondria-dependent cascade, resulting in mitochondrial dysfunction, increased intracellular ROS levels, and the release of Cytc from mitochondria into the cytoplasm, thereby activating caspase-3 (43). AGEs (Advanced glycation end products) promote osteoblast apoptosis and are critical in the pathogenesis of diabetic osteoporosis. Sufibin directly downregulates RAGE (receptor of advanced glycation endproducts) expression, modulates the RAGE-mediated mitochondrial pathway, and prevents AGE-induced osteoblast apoptosis (44). These findings highlight that the protective effects of mitophagy contrast sharply with the destructive impact of AOPPs on mitochondrial integrity.

MOMP enhances the permeability of the mitochondrial outer membrane, facilitating the release of Cyt c, AIF, Smac/DIABLO, cytochrome b5, and mtDNA. Hypoxia triggers apoptosis through caspase activation, which is accompanied by the release of mitochondrial cytochrome C in MC3T3-E1 osteoblasts (45). miR-181a-5p targets Runx1, a transcription factor that negatively regulates AIF-1 expression. The downregulation of Runx1 counteracts the osteogenic differentiation promoted by miR-181a-5p inhibitors, while downregulation of AIF-1 reverses the inhibition of osteogenic differentiation induced by miR-181a-5p mimics (46). The apoptosis of osteoblasts and osteocytes associated with androgen action in bone is linked to an increased Bax/Bcl-2 ratio (47).

P53-dependent MPTP opening is essential for Dex (dexamethasone)-induced osteoblast death. In MC3T3-E1 osteoblasts, Dex-induced MPTP opening is characterized by a decrease in MMP, the formation of mitochondrial complexes between CyPD and ANT-1, and the release of Cytc. The MPTP inhibitor SfA significantly inhibits Dex-induced MMP loss, Cyt c release, and subsequent MC3T3-E1 cell death (48). In osteoblasts exposed to angiotensin II, protein and RNA levels of mitochondrial catalase and MnSOD (manganese superoxide dismutase) were reduced. Angiotensin II suppresses SIRT1 protein levels, leading to hyperacetylation of FoxO3a and inhibition of catalase and MnSOD expression. This uncovers a new SIRT1-FoxO3a-MnSOD pathway involved in mitochondrial oxidative stress and mtDNA damage caused by angiotensin II in osteoblasts (49). Nicotine significantly diminishes MnSOD activity, while Sirt3 enhances it through the deacetylation of MnSOD. Mn (III) tetrakis (4-benzoic acid) porphyrin (MnTBAP, an MnSOD analog) markedly mitigates the detrimental effects of nicotine on osteoblasts. The Sirt3-MnSOD pathway has been recognized as a key regulator of mitochondrial oxidative stress and mtDNA damage caused by nicotine, with MnTBAP showing promise as a potential treatment for osteoporosis (50).

Antimycin A treatment reduces CL peroxidation, allowing actin to play a significant protective role in osteoblasts by inhibiting CL (51). TiO2 (Titanium dioxide) nanoparticles, widely utilized in various engineering and bioengineering applications, enhance superoxide anion production and disrupt antioxidant systems in human osteoblasts (52). Ras induces the expression of ERK-dependent angiogenic transcription factors HIF-1α and VEGF-A in osteoblasts exposed to superoxide activation shock waves (53). Glycolysis supplies 80% of the energy required by osteoblasts under aerobic conditions, with lactic acid being the primary glucose metabolite in bone. During osteoblast maturation, mitochondrial respiration is diminished, and Me2 directs glucose carbon into the malate-aspartate shuttle to sustain glycolysis (54). Citrate, the predominant component of vertebrate bones, plays a crucial role in normal bone development and maintenance by stabilizing precursors and inhibiting their conversion to hydroxyapatite, which results in mineral deposition with smaller size and lower crystallinity (55). WNT3A enhances aerobic glycolysis by elevating the levels of essential glycolytic enzymes, a process referred to as the Warburg effect. This metabolic regulation relies on LRP5 rather than β-catenin and is facilitated by mTORC2-AKT signaling downstream of RAC1. Blocking WNT3A-induced metabolic enzymes hinders osteoblast differentiation *in vitro (* 56). Interruption of intra-mitochondrial symbiosis affects osteoblast function through various mechanisms, including energy metabolism dysregulation, impaired cell signaling, and increased oxidative stress, potentially exacerbating bone-related diseases such as OP. Understanding mitochondrial regulatory mechanisms in osteoblasts could illuminate the pathogenesis of bone diseases and identify potential targets for novel therapeutic strategies.

### 3.2 Interference in mitochondrial intersymbiosis with osteoclasts

Osteoclasts are multinucleated cells primarily responsible for bone resorption, breaking down the bone matrix to release minerals, thereby maintaining the balance of bone remodeling. Impairment of mitochondrial function can disrupt the differentiation process of osteoclasts, potentially inhibiting their development and, consequently, affecting the normal process of bone remodeling. This section primarily explores the connection between the intracellular signaling pathways resulting from the interruption of mitochondrial symbiosis and their implications for osteoclasts.

NFATc1 is a crucial transcription factor involved in osteoclast differentiation and is essential for the regulation of osteoclast-specific genes. The ITAM collaborates with FcRγ and DAP12-associated immunoglobulin-like receptors to facilitate NF-κB receptor activator (RANKL) co-stimulatory signaling, subsequently activating calcium signaling via PLCγ (57). Mitochondrial calcium (Ca²^+^) cycling facilitates the activation of the transcription factor NFAT (58). In osteoclasts, mitochondrial ROS contribute to apoptosis (59). Dysfunction in Cyt c oxidase enhances macrophage phagocytosis and promotes osteoclast formation (60). Phosphorylation of BCL2 is involved in the autophagy and differentiation of osteoclasts from OCPs induced by RANKL. RANKL initiates autophagy in OCPs through phosphorylation of BCL2 at the S70 site, which enhances osteoclastogenesis. This suggests that targeting the inactivation of BCL2 at the S70 site in OCPs may offer a therapeutic strategy for addressing pathological bone loss (61). Curcumin blocks RANK signaling and the subsequent JNK-BCL2-Beclin1 pathway, effectively reducing RANKL-induced autophagy in OCPs (62). In Tfam cKO mice, levels of Tfam, mtDNA copy number, and ATP in osteoclasts are significantly decreased. While Tfam cKO mice are smaller than the control group, their trabecular bone volume does not change despite the lack of Tfam. Histological examination of the proximal tibia and lumbar vertebrae shows a notable decrease in the number of osteoclasts in Tfam cKO mice. Despite a pro-apoptotic tendency in Tfam cKO osteoclasts, their bone resorption activity is increased (63).

Recent studies have increasingly highlighted the pivotal role of mitochondrial dynamics in regulating cellular functions, particularly in osteoclasts. Mitochondrial fusion and the dynamic regulation of the mitochondrial network, mediated by fusion proteins such as MFN1 and MFN2, are critical for energy production, cell survival, and numerous intracellular signaling pathways, including calcium homeostasis. Knockout of MFN1 and MFN2 in osteoclast precursors of mice has been shown to increase bone mass in young female mice and prevent the differentiation of osteoclast precursors into mature osteoclasts *in vitro*. Notably, female mice deficient in MFN2 exhibited increased bone mass after one week and showed resistance to osteolysis induced by the receptor activator of NF-κB ligand (RANKL) at 8 weeks. Restoration of osteoclast differentiation by reintroducing intact MFN2 or defective mitochondrial autophagy variants was associated with enhanced calcium entry and normalization of NFATc1 expression, suggesting that MFN2 plays a critical role in controlling the mitochondria-endoplasmic reticulum crosstalk within osteoclasts. This underscores the essential role of MFN2 in maintaining osteoclast function and bone homeostasis (64).

Mitochondrial complex I activity reduces inflammation and promotes bone resorption by influencing the polarization of macrophages into osteoclasts (65). Estrogen deficiency speeds up bone resorption and plays a role in the onset of OP. RANKL triggers complex I activity in OCPs, enhancing OXPHOS and mitochondrial ATP production within three hours of exposure. The effects of mitochondrial bioenergetics are associated with an elevated ability to oxidize substrates from the TCA cycle, along with fatty acids and amino acids (66). Osteoclasts, which are bone-resorptive multinucleated cells derived from monocyte/macrophage lineages, play a crucial role in both normal and pathological bone turnover. Alpha-tocopherol succinate (αTP-suc) has demonstrated potent anticancer activity *in vitro* and *in vivo (* 67). The Warburg phenomenon remains essential for providing a clearer understanding of metabolism and bone remodeling. Inducing the Warburg effect to promote collective glucose metabolism could enhance cell proliferation. While leveraging the energy advantages of the Warburg effect presents challenges, it appears that targeting osteocalcin induces phenotypic changes by linking WNT, Notch, AKT, and insulin signaling pathways, thereby enhancing its efficacy in osteoblast phenotypes. Osteocalcin modulates ATP utilization through the SOST (sclerostin protein) gene within the bone microenvironment. Specifically activating ATP production during the maturation of osteoblasts is an important approach to combat OP (68). In cell biology, the mitochondrial endosymbiosis theory posits that mitochondria originated from an endosymbiotic relationship between primitive eukaryotic cells and archaea. However, interruption of this endosymbiosis can severely impair cellular function. In osteoclasts, the loss of mitochondrial function diminishes their bone resorption capacity. As the cells responsible for degrading bone matrix during bone remodeling, osteoclasts rely on mitochondrial energy to sustain their activity. When mitochondrial function is compromised, osteoclast activity declines, potentially leading to bone metabolic diseases such as osteoporosis. Therefore, maintaining proper mitochondrial function is vital for bone health.

### 3.3 Interruption of mitochondrial endosymbiosis with bone marrow mesenchymal stem cells

Interruption of mitochondrial endosymbiosis profoundly impacts the function and differentiation potential of BM-MSCs (bone marrow mesenchymal stem cells). BM-MSCs possess multidirectional differentiation potential, allowing them to differentiate into osteoblasts, adipocytes, and chondrocytes. Mitochondria serve not only as the core of energy metabolism in BM-MSCs but also regulate critical biological processes, including cell proliferation, differentiation, apoptosis, and autophagy. BM-MSCs mitigate hypoxia-induced mitochondrial damage and apoptosis, possibly through upregulation of Mfn2 expression in mouse trophoblast cells, which alters mitochondrial structure (69). Pinocytosis, a process vital for the removal of dead and dying cells, is essential for maintaining tissue homeostasis. However, pinocytosis impairs osteoblast differentiation, as transcriptional analysis and functional assays reveal that mitochondrial function in MSCs is downregulated following pinocytosis. Experimentally, pinocytosis induced mitochondrial fission in BM-MSCs, and pharmacological inhibition of this fission reduced cell activity and preserved osteoblast differentiation, indicating that pinocytosis-mediated mitochondrial remodeling is crucial for BM-MSC differentiation (70). Bcl-Xl is expressed at comparable levels in both undifferentiated and differentiated hMSCs, while Bcl-2 expression is limited to differentiated cells. The downregulation of Bcl-Xl in undifferentiated hMSCs leads to increased sensitivity to apoptosis, whereas the overexpression of Bcl-2 triggers apoptosis (71). BMSC (Bone marrow stromal cells)-BMP2 induces GSC apoptosis, inhibits proliferation, reduces the formation of GSC neurospheres and their diameters, and downregulates the expression of GSC markers Nanog and OCT4, indicating stemness inhibition (72). Activation of AT1R signaling stimulates BM-MSC apoptosis, linked to increased mtROS production and reduced mtDNA content (73). Mitochondrial dysfunction is a defining feature of numerous tissue injuries and the aging of stem cells. Although the tissue-repairing properties of MSC-EVs (mesenchymal stem cell-derived extracellular vesicles) are well established, the TFAM-OE (overexpression of TFAM) amplifies the protective benefits of MSC-EVs against mitochondrial damage and inflammation. Studies indicate that MSC-EVs have potential for addressing diseases associated with mitochondrial dysfunction, with TFAM signaling playing a vital role in sustaining their regenerative abilities (74). MSCs (Human mesenchymal stem cells) have the ability to transfer mitochondria to cells with significant impairments. However, this transfer has not been seen in cells with pathogenic mtDNA mutations, like A3243G mutations or 4,977 bp deletions, and is restricted to situations where mitochondrial function is nearly absent. Gaining insight into the mechanisms behind mitochondrial transfer may facilitate the development of cell therapy for mitochondrial repair or gene therapy aimed at treating diseases linked to mitochondrial dysfunction (75). MSC transplantation alleviates metabolic defects in recipient cells through intercellular mitochondrial transport (IMT). However, due to reduced mitochondrial CL content, MSC-derived osteoblasts (MSC-OBs) fail to sequester damaged mitochondria into LC3-dependent autophagy, the presumed mitochondrial autophagy receptor in MSCs. Consequently, the potential of MSC-OBs to rescue metabolic defects and prevent cell death in stress-induced epithelial cells is diminished. Small molecule screening identified PQQ (pyrroloquinoline quinone) as a regulator of mitochondrial autophagy and IMT. Long-term culture of MSC-OBs with PQQ (MSC-ObPQQ type) restored CL content, facilitated mitochondrial sequestration into autophagosomes, and activated mitochondrial autophagy (76). NAD/NADH reoxidation modifies the *in vitro* metabolic reconfiguration, rejuvenating aging MSCs. Niacinamide treatment increases intracellular NAD^+^ levels, rebalances the NAD^+^/NADH ratio, enhances Sirt-1 activity in high-passage hMSCs, partially restores mitochondrial function, and rejuvenates aging hMSCs. In contrast, human fibroblasts exhibit limited aging due to a relatively stable NAD^+^/NADH balance during expansion (77, 78). When intermitochondrial symbiosis is disrupted, the energy metabolism of BM-MSCs is compromised, leading to a significant decline in cell proliferation and differentiation. This dysfunction can impair bone tissue repair and regeneration, potentially resulting in conditions such as osteoporosis or delayed fracture healing. Therefore, maintaining mitochondrial function stability is crucial for the proper functioning of BM-MSCs and is a key factor in bone tissue engineering and regenerative medicine.

## 4 Is the relationship between the interruption of mitochondrial symbiosis and osteoporosis genetic or adaptive?

The relationship between the interruption of mitochondrial symbiosis and OP involves two primary factors: heredity and adaptation, both of which have garnered significant attention in the biomedical field. Mitochondrial dysfunction is maternally transmitted through genetic pathways, with mtDNA mutations or damage leading to disorders in energy metabolism during OXPHOS, which is closely linked to bone metabolism (79–82). Abnormal mitochondrial function directly impairs osteoblast activity and the bone mineralization process, thereby increasing the risk of OP (83). For example, the AKT-GSK3β signaling pathway regulates OPA1 cleavage associated with mitochondrial dysfunction, which may contribute to OS-induced osteoblast apoptosis (84). Calcium and phosphate supplements promote bone cell mineralization (85).The interruption of mitochondrial function triggers intracellular compensatory responses, leading to oxidative stress and prompting cells to activate protective mechanisms such as the antioxidant response and autophagy (86, 87). While these reactions may support short-term cell survival, they can result in chronic inflammation and ultimately disrupt the balance of bone metabolism over time. Mitochondrial dysfunction reduces overall energy levels, impacts the metabolic processes essential for maintaining BMD, and exacerbates the progression of OP (88, 89). The interruption of mitochondrial symbiosis is not only a direct consequence of genetic factors but also an adaptive response to environmental changes and insufficient energy supply, manifesting in two interrelated scenarios.

## 5 Interruption of mitochondrial symbiosis and inflammation in osteoporosis

The interruption of mitochondrial symbiosis is intricately linked to inflammatory responses in OP, highlighting the complex interplay between bone health and the immune system. Mitochondrial dysfunction results in the excessive production of ROS within cells, leading to oxidative stress (90). Accumulation of ROS directly damages bone cells, activates inflammatory signaling pathways, and fosters a chronic inflammatory response (91). This overproduction of inflammatory mediators, such as TNF-α (tumor necrosis factor α), IL-6 (interleukin-6), and NF-κB (nuclear factor κB), stimulates osteoclast activation and proliferation, thereby accelerating bone resorption (92, 93). The breakdown of mitochondrial symbiosis influences bone cell survival by modulating autophagy and apoptosis, which in turn exacerbates the inflammatory response. interruption of intra-mitochondrial symbiosis causes mitochondria to release Cyt c, initiating apoptosis and ultimately leading to cell death. Whether there is a distinct Cyt c pool within mitochondria specifically involved in apoptosis activation, or if chemically modified Cyt c drives apoptosis, remains unresolved. The production of H_2_O_2_ (hydrogen peroxide) increases before the release of Cyt c, and both events occur prior to apoptosis. The addition of mitochondrial uncoupling agents prevents the formation of H_2_O_2_ and subsequent apoptosis, underscoring the critical role of mitochondria-derived H_2_O_2_. Capturing hydroxyl radicals derived from H_2_O_2_ reduces cell apoptosis. Cytoplasmic Cyt c originates from a single mitochondrial compartment, supporting a unified pool involved in both respiration and apoptosis, and is chemically identical to its natural state (94). Mitochondria generate hydrogen peroxide (H_2_O_2_), which leads to oxidative damage and initiates anti-parasitic and antibacterial responses, ultimately causing cell death. In cultures of primary human bone marrow cells, H_2_O_2_ stimulated the concentration-dependent activation of TRAP-positive multinucleated giant cells and expanded the pit area in osteoclast activity assays. Additionally, H_2_O_2_ promotes the expression of M-CSF (macrophage colony-stimulating factor) and RANKL, while increasing the RANKL/OPG ratio, reinforcing the idea that oxidative stress is linked to enhanced bone resorption and decreased bone mass in healthy women (95). In conclusion, the intricate relationship between mitochondrial symbiosis interruption and inflammatory responses in osteoporosis underscores the critical connection between bone health and immune function.

The interruption of mitochondrial symbiosis triggers the stimulation of RANKL and M-CSF, which may play a crucial role in human osteoclast formation. The identification of RANKL as a central regulator of osteoclast activity has opened new avenues for therapeutic intervention. Denosumab, a highly specific anti-RANKL antibody, has been shown to rapidly and significantly reduce bone resorption. Its pharmacokinetics allow for subcutaneous administration every six months, making RANKL inhibition a therapeutic approach for OP and related conditions (96). CSF1R (Colony-stimulating factor 1 receptor), also referred to as c-FMS, is a receptor tyrosine kinase that interacts with M-CSF and IL-34 as its ligands (97). M-CSF attaches to the cell surface receptor c-Fms, triggering a signaling cascade that promotes osteoclast differentiation. When TNF-responsive stromal cells are present, TNF-α boosts M-CSF gene expression. Subsequently, M-CSF activates the RANK in OCPs. Reflecting the proliferative and survival-promoting effects of M-CSF, TNF-α increases the number of OCPs when stromal cells expressing TNF receptors are present. Targeting the M-CSF signaling pathway offers a therapeutic strategy against TNF-α, as demonstrated by the complete prevention of osteolysis in mice treated with anti-c-Fms after TNF injection, with minimal impact on macrophage numbers. Therefore, M-CSF and its receptor c-Fms emerge as promising therapeutic targets in the context of inflammatory bone erosion (98).

**Figure 2:**
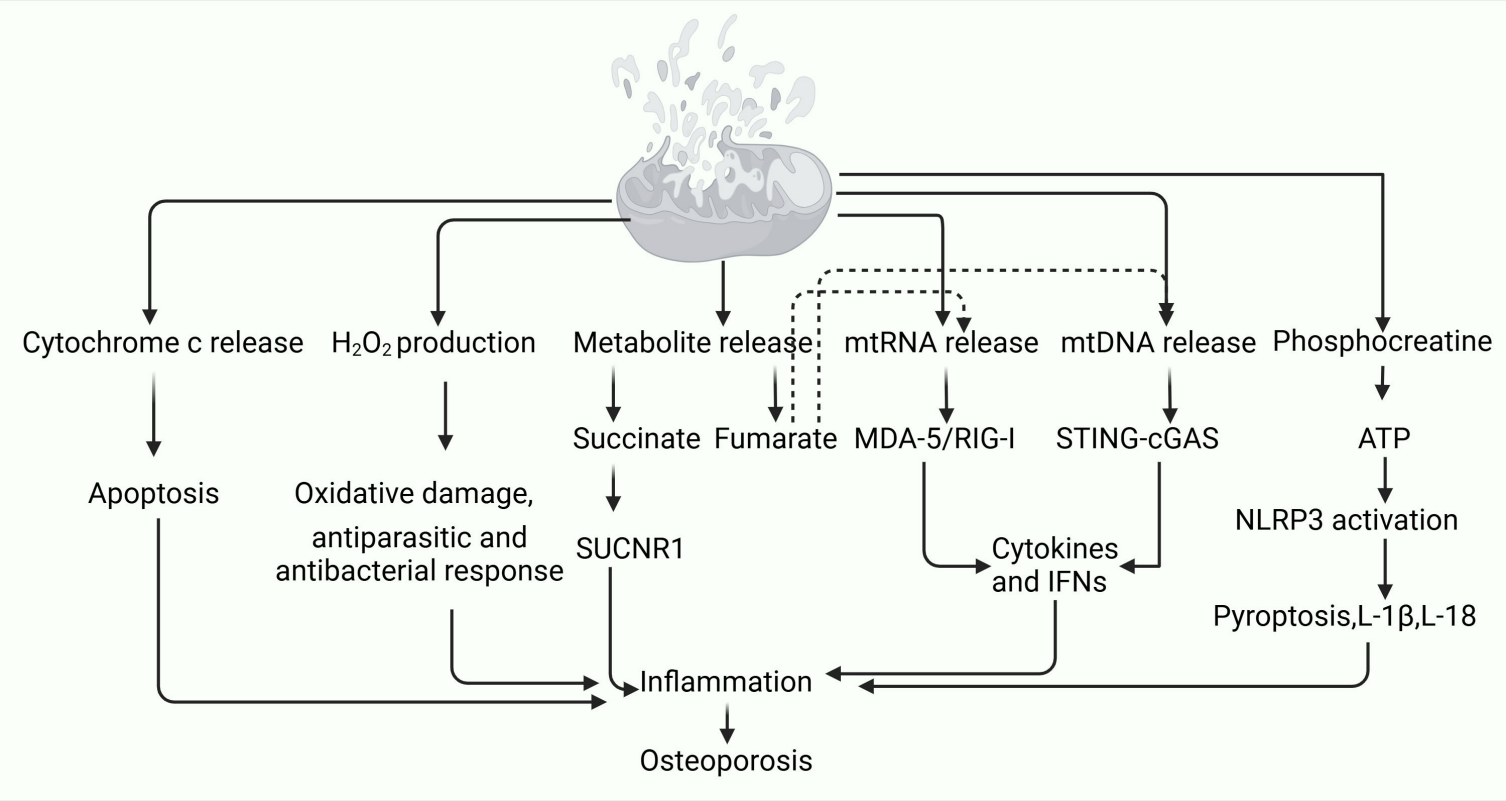
Breakdown of endosymbiosis mediates the mechanism by which inflammation leads to osteoporosis.

The interruption of intra-mitochondrial symbiosis leads to the release of metabolites such as succinate and fumarate, which heighten inflammation through the SUCRN1 pathway. Succinate levels can increase 24-fold during the TCA cycle, acting as an extracellular ligand that promotes osteoclast formation by binding to specific receptors on osteoclast lineage cells. Targeting receptor activation has proven effective in inhibiting osteoclastogenesis (99). Dimethyl fumarate mitigates ROS signaling by boosting antioxidant responses, thereby suppressing osteoclast activity (100). Additionally, the interruption of mitochondrial symbiosis results in the release of mtRNA and mtDNA, which activate the MDA-5/RIG-I and cGAS-STING signaling pathways, leading to cytokine and IFN (interferon) production and triggering inflammation. The release of phosphocreatine activates ATP and NLRP3, inducing pyroptosis and the production of IL-1β and IL-18, further exacerbating inflammation. Sting deficiency has been shown to promote osteoclast to genesis and inhibit osteoblast genesis, with accompanying increases in p-38 phosphorylation in osteoclasts and reductions in β-Catenin phosphorylation in BMSC. While cGAS gene deletion does not significantly affect BMD, Sting gene deficiency has been linked to reduced bone formation in mice, presenting a novel target for OP treatment (101) (**Figure 2**). The breakdown of mitochondrial symbiosis is closely associated with the inflammatory response in OP. Mitochondria, being the central hubs of cellular energy metabolism, when dysfunctional, lead to elevated oxidative stress, which in turn triggers the release of inflammatory factors. In the context of OP, chronic inflammation is recognized as a key factor contributing to bone loss. The interruption of mitochondrial symbiosis results in the excessive release of inflammatory mediators, intensifying osteoclast activity, increasing bone resorption, and ultimately precipitating or exacerbating osteoporosis. Therefore, maintaining mitochondrial function and inhibiting the inflammatory response could emerge as promising strategies for the prevention and treatment of osteoporosis.

## 6 Interruption of mitochondrial endosymbiosis as a potential therapeutic target in osteoporosis

Targeting the interruption of mitochondrial endosymbiosis as a therapeutic approach for osteoporosis underscores the crucial roles of cellular energy metabolism, oxidative stress regulation, and apoptotic pathways in maintaining bone health. Osteoporosis, a metabolic bone disease marked by reduced BMD and heightened fracture risk, is closely linked to decreased bone formation, increased bone resorption, and dysfunction of bone cells. The interruption of mitochondrial symbiosis is a central factor in these pathological processes. A comprehensive understanding of the related molecular targets and the development of targeted therapies are of paramount importance for the effective treatment of OP.

Osteocyte apoptosis, triggered by various factors, plays a pivotal role in the pathogenesis of OP. Modulating bone metabolism disorders by inhibiting the apoptosis of osteoblasts and osteocytes, or by inducing apoptosis in osteoclasts, is fundamental to OP therapy. Therapeutic agents targeting mitochondrial symbiosis and disrupting apoptosis pathways include Gastrodin, Sulforaphane, Crocin, Chlorogenic acid, Luteolin, Geniposide, Curcumin, Tabersonine, Icariin, Grape seed proanthocyanidins, Naringin, and Vanillin, among others. Additionally, **Table 1** provides a list of potential therapeutic drugs aimed at targeting the interruption of intramitochondrial symbiosis in OP.

**Table 1:** Interruption of mitochondrial symbiosis as a potential therapeutic target and drug for osteoporosis.

Drug	Mechanism	Drug regulation mechanism	Refs.
metformin	Mitochondria are involved in immune signaling and programmed cell death	Promote AMPK/SIRT1/NF-κB signaling to drive scorch death of cancer cells	(102)
curcumin	Mitochondria are involved in immune signaling and programmed cell death	Regulate the immune system and release inflammatory mediators, eliminate oxygen free radicals, decrease cell apoptosis, and enhance mitochondrial dynamics to restore kidney function.	(103)
Venetoclax, inhibitor of BCL-2	Mitochondrial outer membrane permeates	Blocking BAX/BAK driven MOMP by upregulating MCL-1 and BCL-XL	(104)
ABT-737	Mitochondrial outer membrane permeates	Induced oligomerization of Bax and/or Bak monomers inserted into the mitochondrial membrane caused Bax, Bak, and Bim to detach from Bcl-2 and Bcl-xL, while remaining associated with Mcl-1L.	(105)
Shikonin	Mitochondrial outer membrane permeates	interruption of ER stress-mediated Caspase-3-targeted tumor apoptosis and Bax/Bak-induced mitochondrial outer MOMP leading to cancer cell apoptosis.	(106)
mPTP small molecule inhibitor GNX-4728	MPTP	Increase mitochondrial calcium retention	(107)
Sanglifehrin A (SfA), an inhibitor of mPTP	Mitochondrial Permeability transition pore (MPTP)	Inhibiting the opening of mPTP prevented cell damage and death caused by dexamethasone (Dex)	(108)
Manganese (III) tetra-(4-benzoate) porphyrin (MnTBAP, a MnSOD analog)	mtDNA	Simulates the function of MnSOD to counteract mitochondrial oxidative stress and mtDNA damage caused by nicotine.	(49)
Notoginsenoside R1 (NGR1)	Mitochondrial DNA (mtDNA)	Reduce cell apoptosis, restore osteoblast activity and osteogenic differentiation ability; Reduce the production of oxidative stress-induced mitochondrial ROS, restore the production of MMP, ATP, and mtDNA copy number.	(51)
Resveratrol	Mitochondrial DNA (mtDNA)	Enhanced mitochondrial biosynthesis during osteogenic differentiation of PO-MSCs, including mtDNA replication and mitochondrial increase.	(109)
IMTs(Inhibitors of Mitochondrial Transcription)	Mitochondrial RNA (mtRNA)	Effectively impairs mtDNA transcription in recombinant systems and leads to dose-dependent inhibition of mtDNA expression and OXPHOS in cell lines	(110)
Adriamycin (ADM) and its derivatives	Function and importance of cardiolipin (CL)	It shows high affinity for cardiolipin (CL)	(111)
Betulinic acid (BetA)	Function and importance of cardiolipin (CL)	The morphological changes of HeLa cell mitochondria were induced. It can quickly and directly affect the saturation level of cardiolipin.	(112)
avasopasem manganese (AVA)	Superoxide produced by the mitochondrial respiratory chain	Antioxidant, anti-inflammatory; Restore the normal function of ETC, reduce the production of ROS, and protect cells from damage	(32)
FeTMPyP (iron porphyrin)	Superoxide produced by the mitochondrial respiratory chain	Increases mitochondrial superoxide levels, but is not affected by oxygen nitrite (PN) or the PN donor (SIN-1)	(113)
Antimycin A and Rotenone	Superoxide generated by the mitochondrial respiratory chain	Blocking various complexes in the mitochondrial electron transport chain disrupts the normal flow of electrons and results in elevated superoxide production within the mitochondria.	(114)
Alpha-lipoic Acid (αLA)	Mitochondrial metabolites act as signals	Promote the production of mitochondrial acetyl-CoA	(115)
Mitochondria-targeted Clonidamine (Mito-LND)	Mitochondrial metabolites act as signals	Inhibition of mitochondrial respiratory chain complex I and decrease of MMP indirectly affect the production and signaling of mitochondrial metabolites	(116)
Triphenylphosphine Modified tripyridine-platinum (II) Complex (TTP)	The Warburg effect and its relationship with mitochondrial function.	It has a strong inhibitory effect on mitochondria and glycolytic bioenergetics	(117)
Mitochondrial targeted monofunctional platinum complex OPT	The Warburg effect and its relationship with mitochondrial function.	It causes notable alterations in the ultrastructure and membrane of the mitochondria, resulting in greater mitochondrial dysfunction compared to cisplatin.	(118)
Gastrodin	Mitochondrial membrane potential	Mitochondrial and ER stress-related signaling pathways regulated by Nrf2 protect osteoblasts	(119)
sulforaphane	Mitochondria-mediated apoptosis	The growth inhibition and release of lactate dehydrogenase in MC3T3-E1 cells induced by dexamethasone were inhibited. Additionally, the mitochondria-mediated apoptotic pathway played a major role in dexamethasone-induced apoptosis, including the activation of caspase-3/-9 and the cleavage of polyadenosine diphosphate ribose polymerase, both of which were effectively prevented by SFP.	(120)
Crocin	Mitochondrial transmembrane potential	Inhibition of ROS/Ca2+ mediated mitochondrial pathway protects osteoblasts from dexamethasone-induced apoptosis	(121)
Chlorogenic acid	Mitochondrial superoxide production	Activation of p21 promotes the Nrf2/HO-1 antioxidant pathway, thereby preventing dexamethasone-induced mitochondrial apoptosis in osteoblasts	(122)
Luteolin	Mitochondrial dysfunction	Activation of PI3K/AKT axis improves mitochondrial dysfunction, alleviates GSDME-mediated pyrodeath, and maintains osteogenesis	(123)
Geniposide	Mitochondria-mediated apoptosis	Reducing endoplasmic reticulum stress and mitochondrial apoptosis in osteoblasts triggered by dexamethasone.	(124)
Curcumin	Mitochondrial dysfunction	Preserve mitochondrial function and activate Akt-GSK3β signaling pathway to improve oxidative stress-induced osteoblast apoptosis	(125)
Tabersonine	Regulation of levels of mitochondrial superoxide and reactive oxygen species	Enhanced cell viability and ALP activity, along with reduced levels of mitochondrial superoxide, reactive oxygen species, matrix metalloproteinases, and osteoblast apoptosis.	(126)
Icariin	Mitochondrial membrane potential	Protect against dysfunction of mitochondrial membrane potential and ROS production due to iron overload, enhance osteoblast survival, and restore the reduced expression of Runx2, alkaline phosphatase, and osteopontin resulting from iron overload.	(127)
Grape seed proanthocyanidins	MMP and respiratory chain complex IV	MMP and respiratory chain complex IV to improve H_2_O_2_-induced mitochondrial dysfunction and reduce mitochondrial free radical production, ROS, and mitochondrial superoxide	(128)
Naringin	Mitochondria-mediated apoptosis	Regulate the activity of mitochondrial apoptosis pathway to promote the apoptosis of osteoclasts	(129)
Vanillin	Mitochondria-mediated apoptosis	The expression of cytochrome c, lytic caspase-3, BAX, and Apaf-1 was upregulated at both mRNA and protein levels, triggering mitochondria-dependent apoptosis.	(130)

The mitochondrial permeability transition, triggered by the opening of MPTP, results in a collapse of bioenergy and subsequent cell death. Edaravone has been shown to inhibit Dex-induced MPTP opening and reduce MMP in osteoblasts. However, the protective effect of Edaravone on Dex-induced osteoblast damage was mitigated when MPTP was inhibited by Cyclosporin A or cyclophiline D siRNA (131). NGR1 was found to decrease the expression of JNK and P-JNK, while simultaneously increasing MMP, mtROS, ATP levels, and mtDNA. It also promoted the levels and activities of ALP, OCN, COLI, and Runx2. NGR1 alleviates mitochondrial dysfunction induced by oxidative stress and restores osteoblast function by inhibiting the JNK signaling pathway. Resveratrol, a natural polyphenol with antioxidant, anti-aging, and anticancer properties, has also shown significant effects in enhancing the osteogenic capacity of BM-MSCs and upregulating mitochondrial biogenesis. Treatment with resveratrol (5μM) increased ALP activity and calcium deposition, indicating enhanced osteogenesis. During the osteogenic differentiation of PO-MSCs, resveratrol treatment (5μM) improved mitochondrial mass and mtDNA copy number, further promoting mitochondrial biogenesis (110).

Although the interruption of mitochondrial intersymbiosis holds potential as a therapeutic target for OP, drug development in this area remains limited. The intricate nature of mitochondrial functions presents significant challenges for the design of mitochondria-targeted therapies. These therapeutic approaches remain largely experimental, with significant challenges persisting, particularly in the development of mitochondria-targeted interventions. Among these, compounds such as metformin, resveratrol, and crocin have been investigated in mitochondrial therapy through randomized controlled trials (RCTs) and prospective studies. While the clinical relevance of metformin is well-established, including its widespread applicability, short-term exposure to clinical doses has been shown to increase skeletal muscle mitochondrial H_2_O_2_ emission and production in healthy elderly individuals (132). In contrast, the therapeutic promise of resveratrol, although supported by earlier *in vitro* studies in mitochondrial myopathy, has not translated into meaningful *in vivo* outcomes. A recent study confirmed the lack of clinical efficacy, and a cross-over RCT demonstrated no significant benefit of resveratrol in patients with mitochondrial myopathy (133).

The fully human monoclonal antibody denosumab, approved in 2010 for the treatment of osteoporosis, has demonstrated robust anti-resorptive effects, leading to significant increases in BMD and reductions in fracture risk at critical skeletal sites. However, it is now evident that transitioning to an alternative anti-osteoporosis therapy following denosumab discontinuation is crucial to mitigate the transient rebound in bone turnover. This rebound effect can cause rapid BMD loss and significantly elevate the risk of multiple vertebral fractures (MVF) (134). Denosumab-related side effects, including hypocalcemia, fracture risk, and potential drug resistance, are particularly relevant in bone diseases where RANKL signaling is a predominant driver. Emerging approaches, such as mitochondrial-targeted therapies, hold promise in this context by modulating osteoclast metabolic activity and viability through the regulation of mitochondrial dynamics, including fusion, fission, and ROS production. Although preclinical studies suggest that mitochondrial-targeted interventions can effectively restore the balance between osteoblast and osteoclast activity within the bone microenvironment, leading to significant improvements in bone density in experimental models, these strategies remain in the early stages of development. Critical gaps persist in understanding their long-term safety and efficacy, and technical hurdles in the design of mitochondria-specific drugs further complicate their clinical translation. While the experimental results are encouraging, extensive research is necessary to establish the feasibility and therapeutic value of mitochondrial-targeted therapies in clinical osteoporosis management.

## 7 Conclusion and prospect

This study highlights the critical association between mitochondrial dysfunction and the pathogenesis of osteoporosis. Disruption of mitochondrial homeostasis undermines the delicate balance of bone metabolism, characterized by impaired osteoblast activity and heightened osteoclast function. This imbalance results in decreased bone density, structural deterioration, and ultimately, the development of osteoporosis. Moreover, the findings underscore the pivotal roles of mitochondrial integrity, energy metabolism, and oxidative stress in the pathological mechanisms underlying osteoporosis. These insights suggest that preserving mitochondrial function could serve as a promising therapeutic strategy for preventing and managing osteoporosis. This research provides a strong theoretical foundation for the development of innovative, mitochondria-targeted therapeutic interventions aimed at maintaining bone health. Mitochondria, often referred to as the “powerhouses” of the cell, are central not only to energy metabolism but also to a wide range of cellular processes. Targeting mitochondrial dysfunction holds significant promise as a therapeutic strategy, yet numerous challenges and limitations remain. A key hurdle lies in the heterogeneity of mitochondrial dysfunction, driven by factors such as genetic variation and environmental influences, which complicates the development of universal treatment approaches. Equally concerning are the limitations inherent in current animal models and the scarcity of robust clinical trial data, which hinder the translation of laboratory findings into viable clinical applications. Recent discoveries have revealed that mitochondria exist in distinct subtypes within cells, each specializing in specific functions. Some are primarily involved in energy production under nutrient-starved conditions, while others are dedicated to biosynthetic processes. This functional specialization underscores the critical need to develop therapies capable of selectively targeting specific mitochondrial populations to maximize efficacy and minimize off-target effects. Addressing these challenges will require innovative approaches to drug design and a deeper understanding of mitochondrial biology to ensure precision in therapeutic interventions. Future studies should continue to focus on the molecular mechanism of mitochondrial intersymbiosis interruption, explore more therapeutic strategies targeting mitochondrial function, and combine the results of multi-disciplinary research to develop more accurate and efficient treatment programs. Additionally, personalized treatment plans for different patient groups also need to be further studied in order to achieve better clinical treatment results. A limitation of this study is that, while mitochondrial dysfunction is linked to the development of osteoporosis, establishing a causal relationship between the two remains challenging. It is still unclear whether mitochondrial destruction directly results in osteoporosis or if osteoporosis itself contributes to the decline in mitochondrial function, necessitating further investigation.
